# Supplementary material for: Comparative multiomics analysis of cell physiological state after culture in a basket bioreactor
Source: Sci Rep. 2022 Nov 23;12:20161. doi: 10.1038/s41598-022-24687-4 (PMC9686226; doi:10.1038/s41598-022-24687-4)
Supplement: Supplementary file 1 — Supplementary Information 1. [file 41598_2022_24687_MOESM1_ESM.zip › raw data/Transcriptomic raw data/6.Enrichment/1.GO/TE1vsTC1/GSEA/TE1/PROTEIN_COMPLEX_OLIGOMERIZATION/PROTEIN_COMPLEX_OLIGOMERIZATION(GO_0051259).html]

Details for gene set PROTEIN\_COMPLEX\_OLIGOMERIZATION(GO:0051259)[GSEA]


|  || Dataset | readcount.sample |
| Phenotype | sample.cls |
| Upregulated in class | TE1 |
| GeneSet | PROTEIN\_COMPLEX\_OLIGOMERIZATION(GO:0051259) |
| Enrichment Score (ES) | 0.48005518 |
| Normalized Enrichment Score (NES) | 1.8582698 |
| Nominal p-value | 0.0 |
| FDR q-value | 0.19883768 |
| FWER p-Value | 0.494 |
Table: GSEA Results Summary

  

Fig 1: Enrichment plot: PROTEIN\_COMPLEX\_OLIGOMERIZATION(GO:0051259)
  
Profile of the Running ES Score & Positions of GeneSet Members on the Rank Ordered List

  

| PROBE | DESCRIPTION   (from dataset) | GENE SYMBOL | GENE\_TITLE | RANK IN GENE LIST | RANK METRIC SCORE | RUNNING ES | CORE ENRICHMENT || 1 | 103220400 | KCNG3 |  |  | 600 | 35.704 | 0.3887 | Yes |
| 2 | 103226786 | KCND2 |  |  | 867 | 4.515 | 0.4294 | Yes |
| 3 | 103220812 | KCNF1 |  |  | 1205 | 3.018 | 0.4494 | Yes |
| 4 | 103238468 | KCNA4 |  |  | 2073 | 2.035 | 0.4343 | Yes |
| 5 | 103247014 | LOC103247014 |  |  | 2371 | 1.907 | 0.4432 | Yes |
| 6 | 103234462 | KCTD15 |  |  | 2391 | 1.899 | 0.4645 | Yes |
| 7 | 103227813 | KCTD6 |  |  | 2579 | 1.828 | 0.4774 | Yes |
| 8 | 103244284 | KCNG1 |  |  | 3208 | 1.664 | 0.4687 | Yes |
| 9 | 103230091 | KCTD3 |  |  | 3731 | 1.569 | 0.4636 | Yes |
| 10 | 103222607 | KCTD1 |  |  | 3770 | 1.562 | 0.4801 | Yes |
| 11 | 103231190 | CRTC3 |  |  | 4317 | 1.483 | 0.4729 | No |
| 12 | 103248102 | KCTD14 |  |  | 5447 | 1.355 | 0.4382 | No |
| 13 | 103242323 | TP53 |  |  | 5681 | 1.332 | 0.4432 | No |
| 14 | 103215505 | KCTD9 |  |  | 5740 | 1.327 | 0.4561 | No |
| 15 | 103245635 | TRPM7 |  |  | 6564 | 1.257 | 0.4339 | No |
| 16 | 103214593 | KCTD12 |  |  | 6578 | 1.255 | 0.4479 | No |
| 17 | 103223121 | HSCB |  |  | 7082 | 1.214 | 0.4396 | No |
| 18 | 103224214 | KCND3 |  |  | 8763 | 1.100 | 0.3772 | No |
| 19 | 103220750 | KCNS3 |  |  | 9876 | 1.030 | 0.3394 | No |
| 20 | 103241894 | TP63 |  |  | 10237 | 1.006 | 0.3350 | No |
| 21 | 103244566 | KCNB1 |  |  | 10352 | 1.001 | 0.3416 | No |
| 22 | 103248106 | KCTD21 |  |  | 10672 | 0.979 | 0.3387 | No |
| 23 | 103237307 | KCNV1 |  |  | 11819 | 0.905 | 0.2980 | No |
| 24 | 103242612 | TNFAIP1 |  |  | 11840 | 0.904 | 0.3076 | No |
| 25 | 103214441 | TRIM13 |  |  | 11976 | 0.896 | 0.3120 | No |
| 26 | 103243061 | KCTD2 |  |  | 12310 | 0.875 | 0.3073 | No |
| 27 | 103239088 | KCTD10 |  |  | 12453 | 0.866 | 0.3110 | No |
| 28 | 103223939 | CRTC2 |  |  | 12794 | 0.845 | 0.3056 | No |
| 29 | 103223265 | KCTD17 |  |  | 12984 | 0.834 | 0.3069 | No |
| 30 | 103230721 | KCTD13 |  |  | 13838 | 0.779 | 0.2778 | No |
| 31 | 103231951 | KCND1 |  |  | 13953 | 0.771 | 0.2817 | No |
| 32 | 103225731 | TP73 |  |  | 14718 | 0.726 | 0.2559 | No |
| 33 | 103219565 | TRPM3 |  |  | 14796 | 0.722 | 0.2609 | No |
| 34 | 103235074 | KCNC3 |  |  | 14879 | 0.715 | 0.2655 | No |
| 35 | 103234193 | CRTC1 |  |  | 15023 | 0.706 | 0.2674 | No |
| 36 | 103231707 | KCTD5 |  |  | 15639 | 0.666 | 0.2476 | No |
| 37 | 103245465 | KCNS1 |  |  | 15944 | 0.644 | 0.2415 | No |
| 38 | 103234709 | SHKBP1 |  |  | 16366 | 0.616 | 0.2298 | No |
| 39 | novel.369 | - |  |  | 16754 | 0.587 | 0.2194 | No |
| 40 | 103219595 | TRPM6 |  |  | 17426 | 0.539 | 0.1956 | No |
| 41 | 103246586 | KCTD7 |  |  | 17492 | 0.532 | 0.1989 | No |
| 42 | 103245946 | TRPM1 |  |  | 17528 | 0.531 | 0.2035 | No |
| 43 | 103233285 | KCTD19 |  |  | 18081 | 0.482 | 0.1844 | No |
| 44 | 103244724 | KCTD16 |  |  | 18490 | 0.441 | 0.1713 | No |
| 45 | 103242292 | KCTD11 |  |  | 19836 | 0.298 | 0.1146 | No |
| 46 | 103224255 | KCNC4 |  |  | 21191 | 0.000 | 0.0540 | No |
| 47 | 103248466 | LOC103248466 |  |  | 21280 | 0.000 | 0.0501 | No |
| 48 | 103235018 | KCNA7 |  |  | 21491 | 0.000 | 0.0407 | No |
| 49 | 103246157 | KCTD8 |  |  | 21837 | 0.000 | 0.0253 | No |
Table: GSEA details
[plain text format]

  

Fig 2: PROTEIN\_COMPLEX\_OLIGOMERIZATION(GO:0051259)
  
Blue-Pink O' Gram in the Space of the Analyzed GeneSet

  

Fig 3: PROTEIN\_COMPLEX\_OLIGOMERIZATION(GO:0051259): Random ES distribution
  
Gene set null distribution of ES for
 **PROTEIN\_COMPLEX\_OLIGOMERIZATION(GO:0051259)**
